# A Retrospective Study Examining Self-Reported Physical Activity Levels Among Asthma Patients Using the Behavioural Risk Factor Surveillance System (BRFSS) Database

**DOI:** 10.7759/cureus.69783

**Published:** 2024-09-20

**Authors:** Paramjit K Saini, Nidhi Laxminarayan Rao, Rutva Jani, Emily P Solarte Zabaleta, Anura Manandhar, Suppraja Soundarrajan, Gayathri Shyam

**Affiliations:** 1 Internal Medicine, Christian Medical College and Hospital, Ludhiana, IND; 2 Internal Medicine, KAP Viswantham Government Medical College, Tiruchirappalli, IND; 3 Internal Medicine, CU Shah Medical College and Hospital, Surendranagar, IND; 4 Internal Medicine, Universidad del Zulia, Zulia, VEN; 5 Internal Medicine, Nobel Medical College, Koshi, NPL; 6 Internal Medicine, California Institute of Behavioral Neurosciences and Psychology, Fairfield, USA; 7 Medicine, Government Medical College, Omandurar Government Estate, Chennai, IND; 8 Internal Medicine, Pravara Institute of Medical Sciences, Sangamner, IND

**Keywords:** asthma, brfss database, demographic factors, exercise, physical activity, united states

## Abstract

Introduction: Regular physical activity benefits respiratory health by reducing the risk of developing asthma. This is achieved by reducing bronchial hyperresponsiveness and preventing lung function decline.

Aim: The objective of the study is to assess the prevalence of self-reported physical activity among asthma patients in the United States in 2021, based on demographic, socioeconomic, and healthcare access variables.

Methodology: The original research study was conducted using the Behavioural Risk Factor Surveillance System (BRFSS) database for the year 2021. Data regarding asthma status, physical activity, age, gender, race, education level, income level, employment status, and time since the last routine check-up were collected.

Results: In the BRFSS study conducted in the USA in 2021, there were 43,6121 participants in total. Of these, 61,362 (14.07%) had asthma and 374,759 (85.93%) did not; 43,678 (71.2%) participants with asthma were engaging in physical activity while 17,684 (28.8%) were not. In the group of participants who did not have the disease, 285,932 (76.3%) were engaging in physical activity and 88,827 (23.7%) were not. Demographically, the highest physical activity among those with asthma was observed in the age group of 18 to 24 years (4,079, 83%), male participants (17,725, 76.4%), and white non-Hispanics (31,964, 72.5%). Higher physical activity levels among asthma patients were associated with advanced education 31,947 (76.5%), employment 23,233 (79.8%), and annual incomes exceeding $150,000, 4,091 (89.9%).

Conclusion: Participants who self-reported not having asthma have a higher prevalence of physical activity in all categories studied. There is a significant association between physical activity and self-reported asthma, shaped by demographic and socioeconomic factors, as well as the frequency of routine medical check-ups.

## Introduction

Asthma is an immune-inflammatory chronic lung disease affecting 8% of the adult population in the United States [1,2]. Several common pathophysiological pathways have been identified, such as genetic predisposition, dysregulated innate immunity, and airway epithelial barrier damage, that contribute to the remodeling of the airway wall. History of prematurity, early lung infections, rhinitis, smoking, and obesity are all risk factors that [1] sustained a complex heterogeneous presentation, severity, and therapeutic response [3].

Physical activity is defined as any body movements produced by the activation of skeletal muscles that lead to a substantial increase in energy expenditure above resting metabolism [4]. Regular physical activity has been shown in several studies to have a positive impact on respiratory health and asthma, reducing the risk of developing the disease by lessening bronchial hyperresponsiveness and decreasing lung function decline [5]. Additionally, it plays a crucial role in preventing complications; it is shown that as the level of physical activity increases, the risk of asthma exacerbations decreases [6]. Although the benefits have been widely identified, some studies have indicated that asthmatic patients engage in lower levels of physical activity in comparison to the general population [7,8]. Physical inactivity is considered a significant modifiable risk factor for impaired respiratory function, asthma management, and quality of life [3]. In this study, the authors aim to assess the prevalence of physical activities in asthma patients based on demographic, socioeconomic, and healthcare access variables using the Behavioural Risk Factor Surveillance System (BRFSS), a tool that collects data about United States residents regarding chronic health conditions, risk factors, and preventive services via telephonic surveys.

## Materials and methods

A retrospective original research study was conducted using the BRFSS Database, with data extracted on April 20, 2024 [9]. Since this is a retrospective study using de-identified, publicly available data, no ethics committee approval was required. The data, from the BRFSS-web-enabled analysis tool (WEAT) for 2021, covers all U.S. states and was selected using various variables. The primary variables in this study are asthma status and physical activity. Asthma status was measured by whether respondents were ever told they had asthma (ASTHMA3). Physical activity was assessed by asking if respondents had engaged in any physical activities or exercises such as running, calisthenics, golf, gardening, or walking for exercise in the past month (EXERANY2).

Control variables were included to adjust for potential confounders in the relationship between asthma status and physical activity. These were demographic, socioeconomic, and healthcare access variables. Demographic variables included age, gender, and race. Age (_AGE_G) was categorized into four groups: 18 to 24, 25 to 44, 45 to 64, and 65 and older. Gender (SEX1) was represented as male or female. Race (_RACEGR3) was classified into four categories: White, non-Hispanic; Black, non-Hispanic; Hispanic; and others.

Socioeconomic variables initially included detailed categories for education level, employment status, and annual income. For education level (EDUCA), the categories were never attended school or only kindergarten, attended grades 1 to 8 (elementary), grades 9 to 11 (any high school), grade 12 or general education development (GED) (high school graduate), college of one to three years (any college or technical), and college 4+ years (college graduate). These were grouped into a new variable with two categories: basic education (never attended school or only kindergarten, grades 1 to 8, grades 9 to 11, grade 12 or GED) and advanced education (college of one to three years, college 4+ years). Employment status (EMPLOY1) was initially categorized as employed for wages, self-employed, out of work for one year or more, out of work for less than one year, and homemaker. These were grouped into two categories: employed (employed for wages, self-employed) and not employed (out of work for one year or more, out of work for less than one year, homemaker). Annual income (INCOME3) categories ranged from $10,000 to over $200,000. These were consolidated into three categories: low-income (less than $50,000), mid-income ($50,000 to $150,000), and high-income (more than $150,000).

The healthcare access variable, CHECKUP1, measures the timing of the last routine check-up. Initially detailed individually, the categories were grouped for clarity. 'Within the past one year' includes check-ups within one to 12 months. 'More than a year ago or never' includes check-ups within one to two years, two to five years, five or more years, and never.

Only data meeting the specified variables and criteria were included in the study. Any data outside these criteria present in the database were excluded. Descriptive data for each variable were generated using cross-tabulations in the BRFSS-WEAT, calculating the number and percentage of responses for various demographic, socioeconomic, and health-related categories. The collected data were then organized and stored in Microsoft Excel (Microsoft Corp., Redmond, WA, USA).

For statistical analysis, R version 4.3.1 (RStudio, Boston, MA, USA) was employed. Relationships between variables were examined using chi-square tests and Fisher’s exact tests. This comprehensive approach provided valuable insights into the physical activity levels among asthma patients, considering their demographic, socioeconomic, and healthcare access characteristics. This allowed for a thorough understanding of the behavior and characteristics of asthma patients in relation to their physical activity levels.

## Results

The BRFSS study of 2021 includes a total of 436,121 U.S. participants. Around 71.2% answered 'yes' to the questionnaire regarding any kind of physical exertion such as walking, running, golf, or gardening (EXERANY2). These participants were considered to have asthma. Table 1 shows the prevalence of self-reported asthma based on physical activity in the study participants.

**Table 1:** Prevalence of self-reported asthma per physical activity level Values are mentioned in n (%).  *A p-value <0.05 is considered significant.

Parameters		During the past month, did you engage in any physical activities, or exercises such as running, calisthenics, golf, gardening, or walking for exercise (EXERANY2)?		p-value (chi-square test)
		Yes	No	
Were you ever told you had asthma?	Yes	43678 (71.2%)	17684 (28.8%)	<0.001*
	No	285932 (76.3%)	88827 (23.7%)	

In the past month, 71.2% of participants with asthma had engaged in physical activity, while 28.8% had not. In the group of participants who did not have the disease, 76.3% were engaged in physical activity and 23.7% were not. According to the chi-square test, there is a statistically significant association between physical activity level and self-reported asthma. Table 2 shows the prevalence of self-reported asthma involving physical activity level based on the demographic characteristics of study participants.

**Table 2:** Prevalence of self-reported asthma by physical activity level based on demographic characteristics of study participants. Values are mentioned in n (%).  *A p-value <0.05 is considered significant.

Variables	Asthma (yes/no)	N (%)	Physically active	Physically not active	p-value (Fisher's exact test)
Age groups					
18 to 24 years	Yes	4912	4079 (83%)	833 (17%)	0.214
	No	20905	17512 (83.8%)	3393 (16.2%)	
25 to 44 years	Yes	16741	13317 (79.5%)	3424 (20.5%)	<0.001*
	No	88933	72738 (81.8%)	16195 (18.2%)	
45 to 64 years	Yes	21371	14487 (67.8%)	6884 (32.2%)	<0.001*
	No	128690	99003 (76.9%)	29687 (23.1%)	
65+ years	Yes	18338	11795 (64.3%)	6543 (35.7%)	<0.001*
	No	136231	96679 (71%)	39552 (29%)	
Gender					
Male	Yes	23204	17725 (76.4%)	5479 (23.6%)	<0.001*
	No	179245	140736 (78.5%)	38509 (21.5%)	
Female	Yes	38158	25953 (68%)	12205 (32%)	<0.001*
	No	195514	145196 (74.3%)	50318 (25.7%)	
Race					
White, non-Hispanic	Yes	44082	31964 (72.5%)	12118 (27.5%)	<0.001*
	No	276970	214977 (77.6%)	61993 (22.4%)	
Black, non-Hispanic	Yes	5219	3371 (64.6%)	1848 (35.4%)	<0.001*
	No	27377	19361 (70.7%)	8016 (29.3%)	
Hispanic	Yes	5457	3634 (66.6%)	1823 (33.4%)	<0.001*
	No	32759	22599 (69%)	10160 (31%)	
Other	Yes	5169	3694 (71.5%)	1475 (28.5%)	<0.001*
	No	28429	21956 (77.2%)	6473 (22.8%)	

The physical activity in participants with self-reported asthma was highest in age groups 18 to 24 years (83%); male gender (76.4%); and White, non-Hispanics (72.5%). There is a statistically significant association between physical activity and asthma for age groups 25 to 44 years, 45 to 64 years, and above 65 years of age, both male and female gender; White, non-Hispanics; Black, non-Hispanic; Hispanic; and other races. Table 3 shows the prevalence of self-reported asthma involving physical activity based on the socioeconomic characteristics of study participants.

**Table 3:** Prevalence of self-reported asthma by physical activity level based on socioeconomic characteristics of study participants. Values are mentioned in n (%).  *A p-value <0.05 is considered significant.

Variables	Asthma	N (%)	Physically active	Physically not active	p-value (Fisher's exact test)
Education level					
Basic education	Yes	19358	11567 (59.8%)	7791 (40.2%)	<0.001*
	No	117191	75650 (64.6%)	41541 (35.4%)	
Advanced education	Yes	41752	31947 (76.5%)	9805 (23.5%)	<0.001*
	No	255520	208805 (81.7%)	46715 (18.3%)	
Employment status					
Employed	Yes	29122	23233 (79.8%)	5889 (20.2%)	<0.001*
	No	193796	157632 (81.3%)	36164 (18.7%)	
Not employed	Yes	31236	19773 (63.3%)	11463 (36.7%)	<0.001*
	No	173975	123097 (70.8%)	50878 (29.2%)	
Annual income					
Low-income	Yes	24665	15251 (61.8%)	9414 (38.2%)	<0.001*
	No	125105	83817 (67%)	41288 (33%)	
Mid-income $50,000 to $150,000	Yes	19573	15706 (80.2%)	3867 (19.8%)	<0.001*
	No	134839	111799 (82.9%)	23040 (17.1%)	
High-income >$150,000	Yes	4550	4091 (89.9%)	459 (10.1%)	0.017*
	No	34083	31017 (91%)	3066 (9%)	

The physical activity in participants with self-reported asthma was highest in participants with advanced education (76.5%), with employment (79.8%), and with an annual income of more than $150,000 (89.9%). There is a statistically significant association between physical activity and asthma in basic and advanced education levels, employed and non-employed, in low-income, mid-income, and high-income. Table 4 shows the prevalence of self-reported asthma involving physical activity based on time since the last routine check-up of study participants.

**Table 4:** Prevalence of self-reported asthma by physical activity level based on time since last routine check-up Values are mentioned in n (%). * A p-value <0.05 is considered significant.

Variables	Asthma	N (%)	Physically active	Physically not active	p-value (Fisher's exact test)
Time since the last routine check-up					
Within the past one year	Yes	48375	33775 (69.8%)	14600 (30.2%)	<0.001*
	No	287103	217226 (75.7%)	69877 (24.3%)	
More than a year ago or never	Yes	12234	9441 (77.2%)	2793 (22.8%)	<0.001*
	No	82741	65364 (79%)	17377 (21%)	

Among participants with routine check-ups within one year, 69.8% with asthma were physically active as compared to 75.7% who were physically active without asthma. This indicates that the difference is statistically significant. Concerning participants with routine check-ups for more than one year, 77.2% with asthma were physically active as compared to 79% who were physically active without asthma. This shows that the difference is statistically significant.

## Discussion

A retrospective study was conducted in April 2024 to assess the prevalence of physical activity levels among asthmatic patients in the U.S. for the year 2021 using the BRFSS database. Several previous studies show that physical activity can potentially benefit asthmatic patients and improve lung function and their quality of life, but patients frequently avoid physical activity due to concerns that it might worsen their asthma [10-12].

In this study, Table 1 illustrates the prevalence of self-reported asthma based on physical activity levels among the participants. In the month prior to administering the questionnaire, 71.2% of participants with asthma engaged in physical activity, compared to 76.3% of those without asthma. This suggests that physical activity levels are lower in patients with asthma compared to those without the condition. A cross-sectional study done in the UK published in 2015 resulted in primarily younger, White, male participants of normal weight exhibiting higher physical activity levels and better asthma-related quality of life [10]. Most asthma patients in this study had low physical activity levels, failed to meet the recommended physical activity guidelines, and experienced a diminished quality of life. Asthmatics spend much less time engaging in moderate-to-intense physical exercise than their non-asthmatic counterparts, according to a 2011 article [13,14].

We depict the prevalence of self-reported asthma concerning physical activity levels, categorized by the demographic characteristics (age, gender, and race) of the study participants. Physical activity among participants with self-reported asthma was highest in the 18 to 24 age group (83%); in males (76.4%); and among White, non-Hispanics (72.5%). Younger age groups are more physically active than older age groups, and physical activity levels decline with age. Males are generally more physically active than females. The data also shows that White non-Hispanics had the highest prevalence of physical activity compared to other races. However, within each subgroup, physical activity levels were lower in asthma patients compared to those without asthma based on demographic characteristics (age groups, gender, and race). A cross-sectional study done in Spain and published in January 2024 shows similar results in terms of demographics. A decreased body mass index (BMI), younger age, male sex, and better self-rated health were all strongly correlated with greater amounts of physical activity. But overall asthma patients are associated with lower physical activity levels [15,16].

We also identified the prevalence of self-reported asthma in relation to physical activity levels based on socioeconomic characteristics. Physical activity among participants with self-reported asthma was low among those with basic education (59.8%) and increased in those with advanced education (76.5%), employment (79.8%), and an annual income of more than $150,000 (89.9%). However, when comparing physical activity levels between asthmatics and non-asthmatics based on socioeconomic factors, asthmatic patients still have lower levels of physical activity compared to those without asthma. In a population-based study in Sweden, self-reported diagnosed asthma was independently linked to economic difficulties but not to educational level [16]. Lifestyle factors did not account for the association between economic difficulties and asthma prevalence.

We also show the prevalence of self-reported asthma in relation to physical activity levels based on the time since the last routine check-up of the participants. Among participants who had a routine check-up within the past year, 69.8% of those with asthma were physically active compared to 75.7% of those without asthma. For participants whose last routine check-up was more than a year ago or who never had a routine check-up, 77.2% of those with asthma were physically active compared to 79% of those without asthma. So overall physical activity levels tend to be lower in asthma patients, whether they had a routine check-up in the last year or more than a year ago/never, as compared to non-asthmatic patients.

In short, participants who self-reported not having asthma exhibited higher levels of physical activity across all categories studied. There is a significant association between physical activity and self-reported asthma, influenced by demographic and socioeconomic factors, as well as the frequency of routine medical check-ups.

Despite the acknowledged advantages of physical activity for individuals with asthma, there are still deficiencies in physicians' understanding of exercise guidelines and counseling practices. Promoting regular physical activity among adults with asthma is supported by a strong link to enhanced asthma quality of life. This implies that maintaining an adequate level of activity could boost overall well-being even for those living with asthma. [17]

Limitations

This study utilized the BRFSS database, which collected information via a telephone survey. Thus, the BRFSS participants who lack access to landlines, do not use landlines or were not at home when they were called could not be contacted or included in the study. Moreover, the BRFSS survey was administered in English, and thus, patients who do not speak or understand the language or who are members of underrepresented groups were also not included. As the study relies on self-reported data, certain factors that one participant may perceive as intense exercise may appear to another as moderate exercise; nevertheless, the study does not assess these variables. Being a retrospective observational study, only association and, not causation or correlation can be established.

## Conclusions

According to the BRFSS dataset, this study revealed significant details about the prevalence of physical activity among asthmatic participants. Asthma emerged as a crucial determinant, with participants who self-reported not having asthma having a higher prevalence of physical activity in all categories studied. These findings highlight the importance of sociodemographic factors in shaping health literacy and the need for tailored approaches. Future studies should determine how physical activity influences the disease course in asthma patients, which will help generate guidelines for physical activity levels in asthma.
